# Supplementary material for: Consistent Biomarkers and Related Pathogenesis Underlying Asthma Revealed by Systems Biology Approach
Source: Int J Mol Sci. 2019 Aug 19;20(16):4037. doi: 10.3390/ijms20164037 (PMC6720652; doi:10.3390/ijms20164037)
Supplement: Supplementary file 1 [file ijms-20-04037-s001.zip › ijms-566768-supplementary-XML/Supplementary Material S2-Reports of Quality control for ten datasets.zip!/Supplementary Material S2-Reports of Quality control for ten datasets/QC_report_for_GSE64913/hm.pdf]

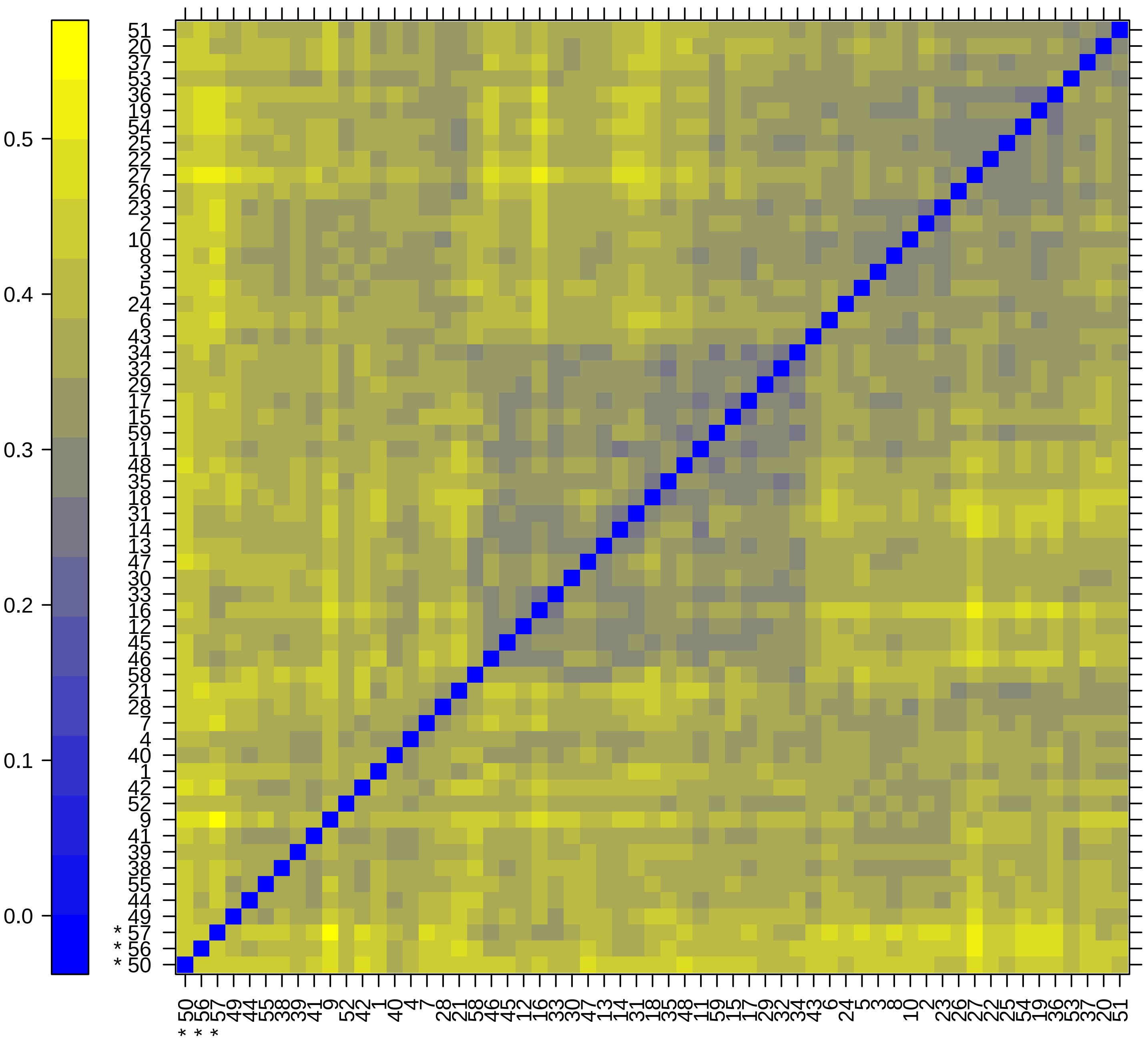
0.0
0.1
0.2
0.3
0.4
0.5
51 20 37 53 36 19 54 25 22 27 26 23 2 10 8 3 5 24 6 43 34 32 29 17 15 59 11 48 35 18 31 14 13 47 30 33 16 12 45 46 58 21 28 7 4 40 1 42 52 9 41 39 38 55 44 49 * 57 * 56 * 50
50 56 57 49 44 55 38 39 41 9 52 42 1 40 4 7 28 21 58 46 45 12 16 33 30 47 13 14 31 18 35 48 11 59 15 17 29 32 34 43 6 24 5 3 8 10 2 23 26 27 22 25 54 19 36 53 37 20 51
* * *
